# Rodent Scope: A User-Configurable Digital Wireless Telemetry System for Freely Behaving Animals

**DOI:** 10.1371/journal.pone.0089949

**Published:** 2014-02-28

**Authors:** David Ball, Russell Kliese, Francois Windels, Christopher Nolan, Peter Stratton, Pankaj Sah, Janet Wiles

**Affiliations:** 1 School of Electrical Engineering and Computer Science, Queensland University of Technology, Queensland, Australia; 2 TOPTICA Photonics AG, Lochhamer Schlag 19, Gräfelfing, Germany; 3 Queensland Brain Institute, The University of Queensland, Queensland, Australia; 4 School of Information Technology and Electrical Engineering, The University of Queensland, Queensland, Australia; Georgia Regents University, United States of America

## Abstract

This paper describes the design and implementation of a wireless neural telemetry system that enables new experimental paradigms, such as neural recordings during rodent navigation in large outdoor environments. RoSco, short for Rodent Scope, is a small lightweight user-configurable module suitable for digital wireless recording from freely behaving small animals. Due to the digital transmission technology, RoSco has advantages over most other wireless modules of noise immunity and online user-configurable settings. RoSco digitally transmits entire neural waveforms for 14 of 16 channels at 20 kHz with 8-bit encoding which are streamed to the PC as standard USB audio packets. Up to 31 RoSco wireless modules can coexist in the same environment on non-overlapping independent channels. The design has spatial diversity reception via two antennas, which makes wireless communication resilient to fading and obstacles. In comparison with most existing wireless systems, this system has online user-selectable independent gain control of each channel in 8 factors from 500 to 32,000 times, two selectable ground references from a subset of channels, selectable channel grounding to disable noisy electrodes, and selectable bandwidth suitable for action potentials (300 Hz–3 kHz) and low frequency field potentials (4 Hz–3 kHz). Indoor and outdoor recordings taken from freely behaving rodents are shown to be comparable to a commercial wired system in sorting for neural populations. The module has low input referred noise, battery life of 1.5 hours and transmission losses of 0.1% up to a range of 10 m.

## Introduction

Since the first recordings of single neurons in anaesthetised animals [1], [2], technological advances have enabled electrophysiological recordings with greater recording precision, less noise and in progressively more natural conditions. Extracellular recordings in animals, made using wire implants within the brain, detect changes in the extracellular voltage when neurons discharge action potentials (APs) or groups of neurons generate low frequency local field potentials (LFPs). Recording from multiple cells simultaneously and discriminating the activity of each cell over time requires high signal to noise recordings at high bandwidth. Moreover, for these recordings to be ecologically significant, animals need to be awake and behaving in natural environments. However, animals are typically tethered to a neural recording system, limiting research to within simple, small indoor environments.

Wireless neural telemetry systems have been in development for decades [3] and are typically designed with particular types of scientific research questions in mind, each with their own requirements and limitations. See [4] for a good review of recent advances and challenges. Our target research involves high fidelity neural recording as one or more rodents perform navigation tasks in outdoor environments. We have identified two complementary sets of criteria that an experimentally-useful wireless solution for outdoor recordings must satisfy: (1) verifiable fidelity – neural recordings must be high fidelity, quantify any interference, record entire waveforms, and permit offline verification of results; and (2) useability – to facilitate practical experiments the channels must be user-configurable, provide sufficient battery power for a complete recording session, and must not interfere with an animal's normal movements. No single rodent neural telemetry device, including currently available commercial solutions, addresses the criteria above including error quantification in noise-prone environments and configurable settings.

In this paper we describe a digital neural telemetry system, Rodent Scope (RoSco), that addresses these criteria. It records 16 channels of neural signals at 8 effective bits, and is a head-mounted module that weighs 22 g and is ideally suited to rodent experiments in outdoor-like environments. Due to the digital design, RoSco has verifiable fidelity and system parameters can be configured in real time. Prior to transmission each channel can be independently grounded to disable noisy electrodes, be amplified in 8 factors from 500 to 32,000 times, and can be filtered for either LFPs or APs. We present results from both our wireless system and an Axona tethered recording system. Both recordings were made in a single session from a freely behaving rat in a laboratory setting, demonstrating similar SNR between the systems and the same number of spike clusters. We also present results from our wireless module from a rat foraging in a 3.5×2.5 m caged outdoor arena. We have made the schematics [5] and firmware [6] for RoSco freely available online to allow other researchers to reuse or modify our design.

Verifiable fidelity is crucial for trust in novel experimental paradigms, and requires measuring the accuracy of the recorded neural signal. Popular commercial solutions such as the Triangle BioSystems W-Series are analog systems. To facilitate experiments in more natural conditions, analogue system require careful attention to remove any possible sources of radio interference that can compromise the integrity of the recording. Analog modules, though lighter and more power efficient than their digital counterparts, cannot quantify transmission noise. Since signal quality is a key requirement in novel experiment settings, we diverged from much of the wireless field in opting for digitisation before transmission. Digitisation also confers other advantages, such as higher spectral efficiency and bi-directional communication as discussed below.

Continual miniaturisation of analog-to-digital and digital transmission components has recently led to the development of a number of digital wireless neural telemetry systems [7]–[14]. The design of these systems varies considerably. Wireless systems can opt to reduce transmission bandwidth requirements by performing spike detection on the wireless module, transmitting only spike times and the spike waveform [10], [14]. However, this design decision can adversely impact later signal analysis. Spike detection is not a simple process as the threshold for detection of single units can affect the classification of these spikes and for many research purposes, complete source waveforms are required for offline analysis.

Usability is a design criterion that covers all aspects of the telemetry system that supports its ease of use in practical experiments by electrophysiologists, and is an essential factor in adoption of new technology. Existing tethered systems have a large set of features to support typical recording tasks. In particular, they allow online, real time configuration of the individual channels, previously recognised as important for a variety of tasks such as detecting and disabling noisy channels, selecting ground reference, and recording at maximum gain without saturation of the signals [7]. The RoSco system has what we consider the minimum set of the online configuration options, including:

- user-selectable independent gain control of each channel in 8 factors from 500 to 32,000 times,
- two selectable ground references from a subset of channels,
- selectable channel grounding to disable noisy electrodes, and
- selectable filters suitable for action (300 Hz–3 kHz) and low frequency (4 Hz–3 kHz) potentials.

Finally, any module must not unduly interfere with the mobility of the animal such that its range of normal behaviour is disrupted, and must operate for long enough to be of practical experimental use. The device therefore is limited in weight and in its possible mounting configurations. Several existing wireless systems for rodent neural telemetry employ a combined head-stage and backpack, together weighing 50 g or more (excluding the weight of the microdrive used for the implants) [10], [12], [14], [15]. However, behaviour can be impacted by the body harness. Smaller and lighter devices can be mounted entirely on the head of the animal with much less impact on the mobility and range of movements of the animal.

### System Description

**Figure 1:**
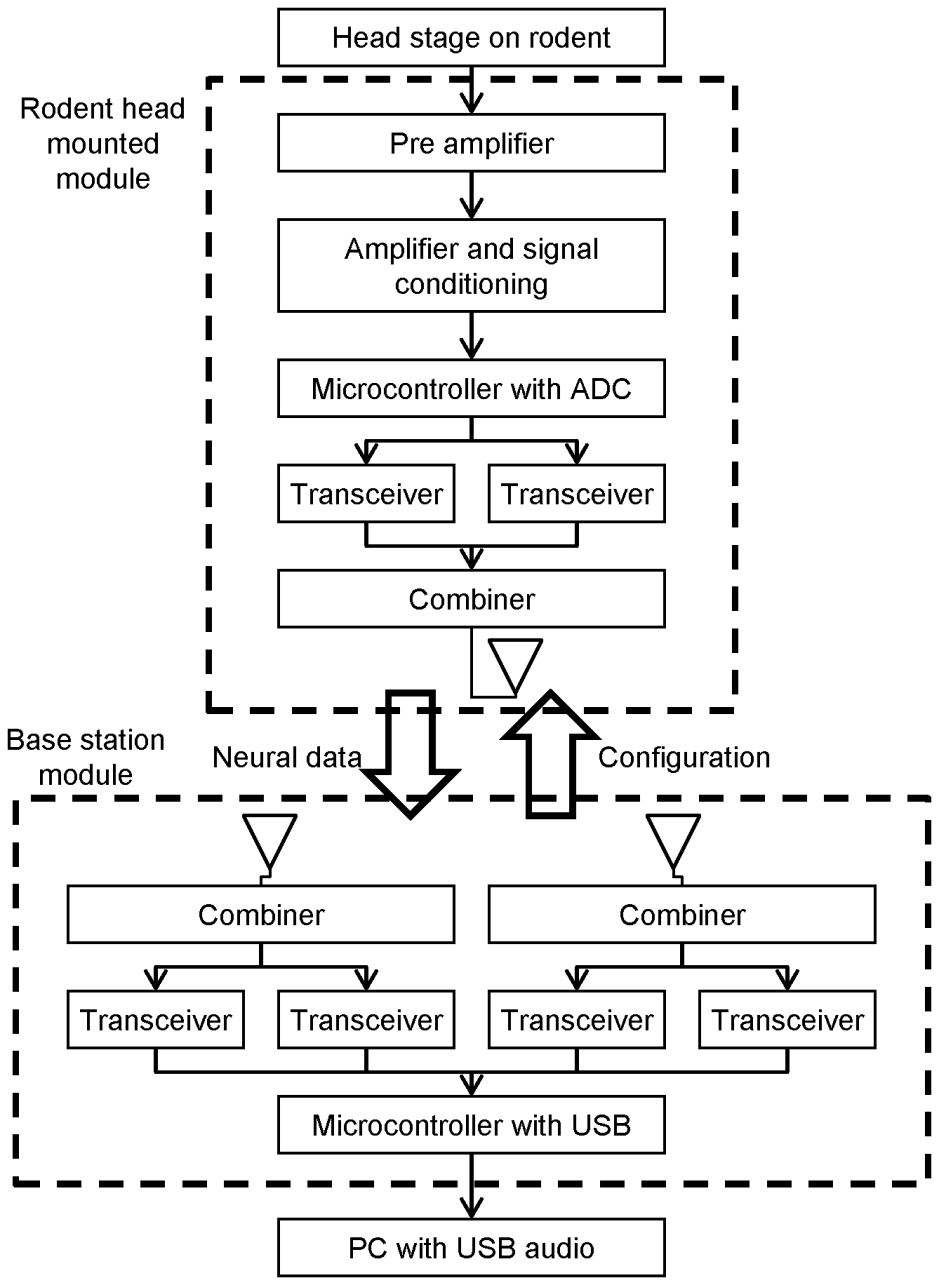
Block diagram of RoSco. The head mounted module connects to the rodent's head stage. The base station module connects to a PC running USB audio software. Bidirectional communication allows transmission of neural data to the PC, and configuration data to the rat mounted module. The RoSco has one communication module which includes two transceivers that simultaneously operate in parallel to transmit the neural waveform through a single combiner and antenna. The base station module has diversity reception with two communication modules that simultaneously receive the same entire neural waveform in parallel. This provides redundancy and if data packets are missing from one stream the base station can still reconstruct the full neural waveform.

This section begins with an overview of how the neural signal is processed followed by a description of each part in detail. A block diagram of the RoSco system is given in Figure 1.

The RoSco system acquires the signal from a head-stage with fixed electrode implants. This signal is first pre-amplified, then filtered using a configurable band-pass filter to capture the band of interest. These pre-amplified signals are then further amplified and digitised. The digitised waveforms are wirelessly transmitted from the head mounted module to the base station. Finally, the base station re-assembles the received waveforms which are streamed to the PC formatted as USB audio packets [16]. To provide immunity to noise and fading the base station uses diversity reception where two pairs of transceivers simultaneously receive the neural data stream in parallel. This means that if data packets are missing from one stream the microcontroller can reconstruct the full neural waveform using the redundant data from the other stream.

The base station transmits neural data to the PC as a USB audio stream, thus no custom operating system drivers are required to operate the device. Module configuration is managed via the USB audio configuration settings. Where possible, RoSco configuration parameters are mapped to conceptually similar USB configuration parameters, such as RoSco gain to USB audio volume. Using a well-supported open standard such as USB audio, opens the potential for interoperability between telemetry systems and user interfaces giving researcher the freedom to customise recording software to fit into their particular experimental workflows.

**Figure 2:**
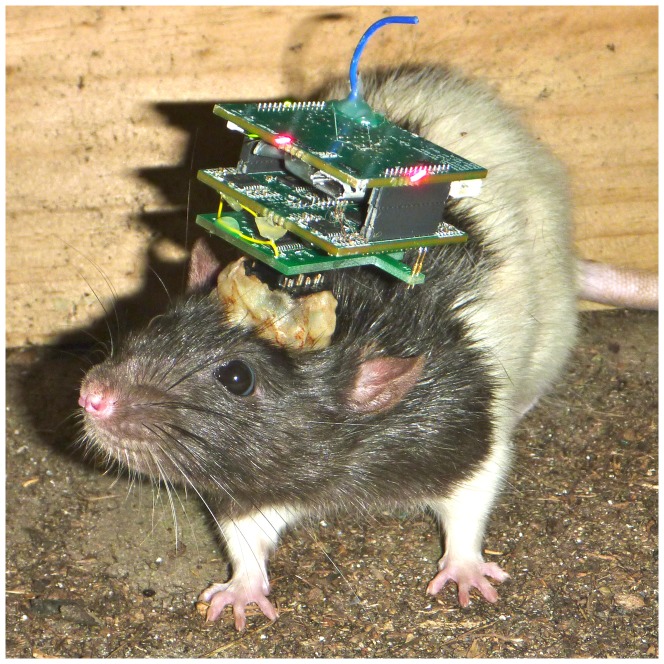
The RoSco head mounted module shown on a Long Evans rat. The head mounted module consists of three PCBs and a small battery. While appearing relatively large in this photo, the head mounted module is light weight. The blue wire is the antenna. The red LEDs are for motion tracking using an overhead camera system.

The system is built entirely from commercially available components populated across four custom printed circuit boards (PCBs). Figure 2 shows a picture of the head mounted module on a Long-Evans rat. The rat head mounted module is composed of three PCBs: a stack of two 35×35 mm PCBs and a smaller PCB that provides the unity gain amplifier stage. Power is provided by a 3.7 V 210 mAh lithium-ion cell weighing 3 g placed between the PCBs. Charging is facilitated by a standard micro-USB socket.

A common method to record the pose of an animal is to track the motion of LEDs. RoSco has four LEDs, one located on each edge of the top PCB (two green and two red). These LEDs can be individually enabled and disabled online.

### Signal amplification and conditioning

**Figure 3:**
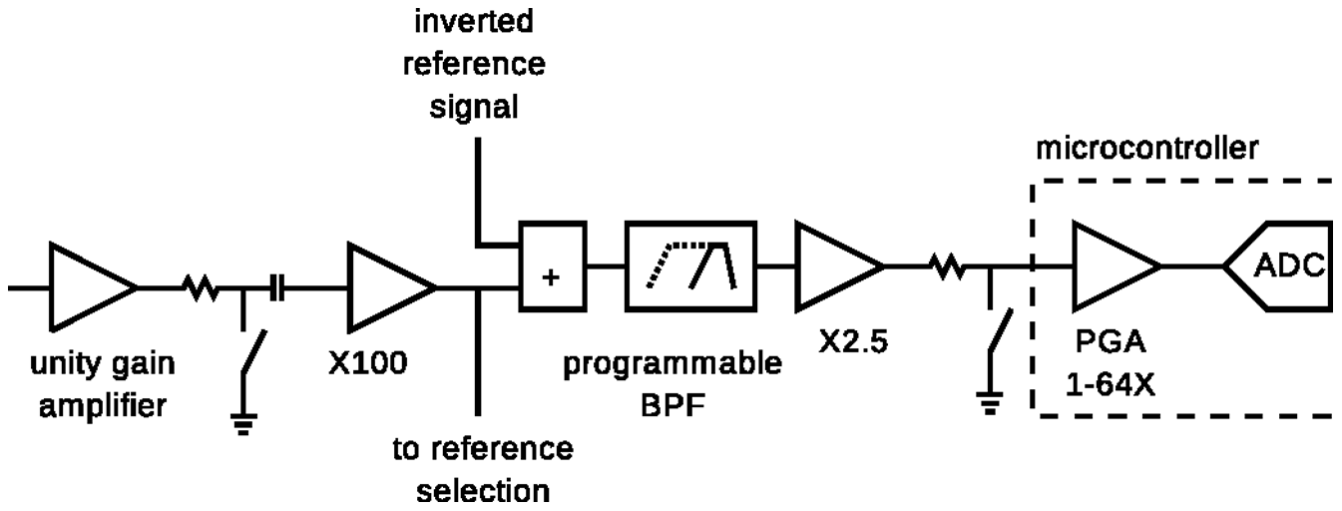
Diagram of one signal amplification and conditioning channel. The diagram shows how the reference, band pass filter and gain can be configured. Note the programmable gain amplifier (PGA) is a part of the microcontroller saving a large number of external components and electronic complexity.

Two frequency ranges are of particular interest in neural recordings: APs in the range 300–3000 Hz (as in [17]) and LFPs at lower frequencies. Filtering is used to remove noise outside the range of interest and amplification is used to boost the signal to a level that can be digitized. The filter's lower cut-off frequency is selectable to allow the acquisition of APs or LFPs. It is only necessary to reduce the lower cut-off frequency to acquire LFP signals because they have higher signal amplitudes [18]. Figure 3 shows a block diagram of one of the 16 analog amplification and signal conditioning stages used in the rat head-mounted module.

The unity gain amplifier is implemented using a low-power, low-noise FET input op-amp (Linear Technology LTC6082) which provides a high input impedance to avoid loading the signal from the recording electrodes. Unity gain, rather than a higher gain, is used to cope with large DC offsets which can be of the order of 1 V [19]. To prevent crosstalk from a noisy input (which may occur when a recording electrode wire breaks) to other channels, the outputs of the unity gain amplifier can be disabled via a digitally controlled analogue switch. Broken wires are relatively common in chronic recordings so the ability to selectively disable channels is essential for practical studies.

The output of the unity gain amplifier is AC coupled to a 100× gain stage (Linear Technology LTC6082) which boosts the signal to provide noise immunity and immunity to cross-talk in subsequent stages. This stage, along with the unity gain amplifier stage, is in close proximity to the electrode connector to reduce the effects of external interference on the weak signals.

After the 100× gain stage a reference signal is added. The 16 input signals are divided into two banks of 8 channels. The reference signal can be chosen from any of the inputs within each bank. This reference signal is then inverted and added to all of the other signals in the bank.

The referenced signal is then passed through an op-amp based (Analog Devices AD8544) active bandpass filter which incorporates an additional 2.5× gain. The lower cut-off frequency has a first-order response and is programmable to cut-off at 4 or 300 Hz. A sharper upper cut-off frequency (third-order) at 3 kHz was implemented to minimize the signal power above 10 kHz that would lead to additional noise (signals above the Nyquist frequency cause aliasing). While it would be possible to design the cut-off frequency closer to 10 kHz, this would be at the expensive of a more complicated filter network with minimal benefit.

### Programmable gain and digitisation

The rat head mounted module has a microcontroller (Atmel ATxmega256A3) with 16 analog to digital converter (ADC) and programmable gain amplifier (PGA) channels. The PGAs were important due to the limited bandwidth which only accommodates 8 bits sample resolution, and the high dynamic range of the neural signals. The high dynamic range is due to the variation in signal strength as the distance between the electrode and spiking neuron changes. The PGAs allows adjusting the neural signal amplitude to cover a large portion of the ADC's limited 8 bit sampling resolution while avoiding signal clipping. The overall gain levels provided are 500, 1000, 2000, 4000, 8000, 16000 and 32000. Lower values are typically used for LFPs and higher values (4000–16000) are typically used for APs. The ADC has a very low Differential Non-Linearity (DNL) of less than ±1 bit.

### Wireless communication

A custom half-duplex wireless communication protocol streams the neural signal from the rat-mounted module to the base station, and sends configuration commands from the base station back to the rat-mounted module. Half-duplex was preferred over full-duplex communication as the configuration data sent back to the rat-mounted module is of very low bandwidth. The bandwidth required for 16 full signal waveforms at 20 kHz with an 8 bit resolution is 2.56 MB/s. Eight bit resolution is adequate for typical neural signals acquired using tetrodes *in vivo* where the signal to noise ratio is typically less than 10∶1 as no additional useful information would be gained from higher resolution. In order to achieve the required bandwidth, a pair of highly integrated ultra-low power half-duplex transceivers (Nordic Semiconductor nRF24L01) communicate simultaneously on separate channels. The pair of transceivers provide a maximum user payload data rate of approximately 3.2 MB/s (after accounting for internal protocol overheads) in simplex operation.

The periodic switching between transmission and reception required to implement half-duplex communication further limits the available data throughput. Even though the configuration data sent back to the rat-mounted module from the PC only occupies one 32-byte packet, significant dead-time limits the total available bandwidth for the neural signal to a value slightly above the 2.56 MB/s required. The cycle time for the half-duplex system is 5 ms. This duration was chosen based on the maximum transmit duration of the Nordic transceivers of 4 ms, after which time the transmit frequency can drift out of tolerance. After the data for one block of 5 ms has been transmitted from the rat-mounted module (which takes approximately 4 ms), the transceivers then listen for commands from the base station until the next block of data is ready to be transmitted. The module configuration options – gain selection, reference channel selection, input grounding and tracking LED toggling – are sent wirelessly to the rat-mounted module from the base station. All configuration settings can be changed while recording is in progress so that the effect of the changes can be observed in real-time.

The radio-frequency output power from each transceiver is 1 mW at 2.4 GHz. On the rat-mounted module the two transceivers are connected to a power combiner to drive a single quarter-wave monopole antenna. The base station has two antennas each connected to their own transceiver pair to provide redundancy in receiving neural data through antenna diversity. This antenna diversity provides immunity to fading such as interference caused when signals arrive from multiple paths due to walls and obstacles.

The modules operate on the international 2.4–2.5 GHz unlicensed industrial, scientific and medical (ISM) band. This provides the two-fold benefits of a fixed band which reduces the need to support a wide range of carrier frequencies and an unlicensed band which avoids the costly approval process required for operation on other bands.

### PC communication

The base station microcontroller (Atmel AT32UC3A3256) reformats the incoming packets from the transceivers into the USB audio protocol. The microcontroller also receives commands from the PC to vary USB audio properties, which are converted into appropriate RoSco commands and transmitted to the head-mounted module.

A digital phase-locked-loop (PLL) provides synchronisation between the head-mounted module and the base-station module. The PLL makes it possible to detect missing packets based on packet timing and to transmit commands to the rat module at the appropriate times. Missing packets are indicated in the USB audio stream using a reserved sample value. (Valid samples containing the reserved missing-packets value are replaced with the next-nearest value.)

## Experimental Procedure

We ran a series of bench tests to measure the performance of the signal conditioning stage including: the bandwidth and gain response, the noise levels, the ground reference selection and the common mode rejection ratio (CMRR). The antenna radiation profile was measured in an antenna range with a vertically polarised horn antenna located in the far-field (∼4 m from the module). The module was mounted on a rotary stage and set to transmit a continuous wave 2.45 GHz signal. The radiation pattern was acquired using a network analyser (HP8530A). The filter transfer functions, CMRR, and noise performance were measured using a National Instruments multifunction data acquisition card (NI PCI-6251, 16 bits/sample, 1 M samples/s). Following bench testing, we measured the wireless performance and battery life of the RoSco system.

**Figure 4:**
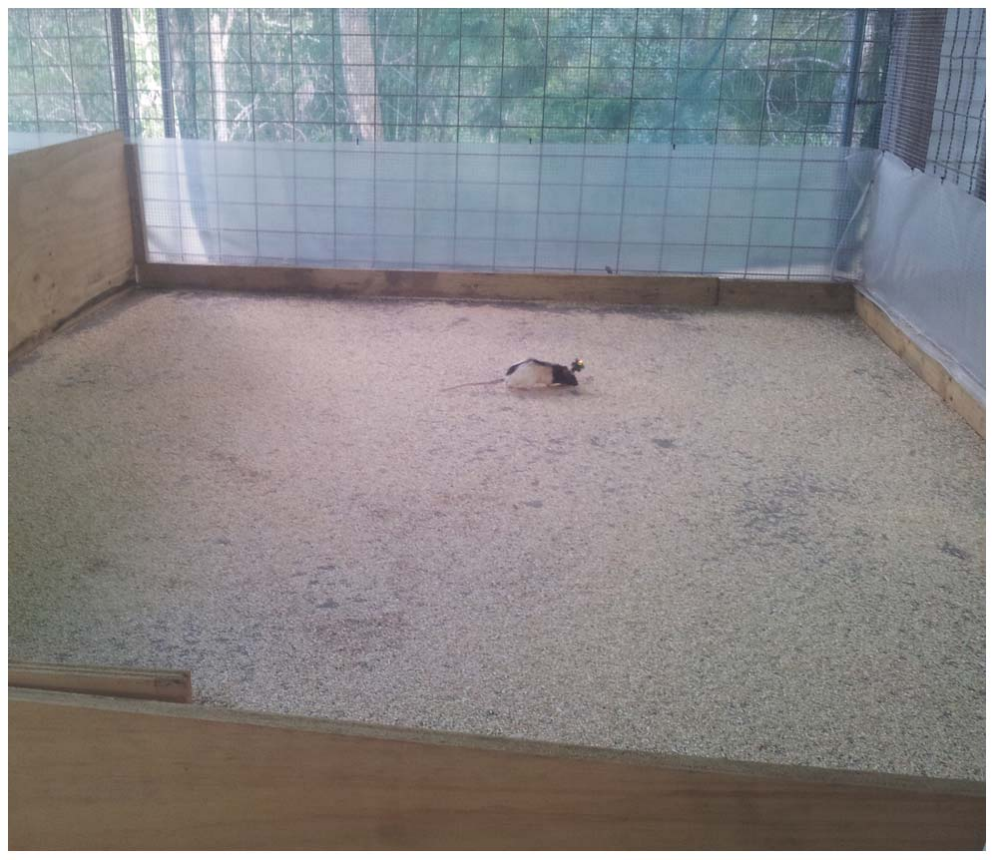
Outdoor rodent test enclosure located in Brisbane, Australia. The enclosure is 7×5 m, has a translucent roof and wire mesh walls. A rodent wearing RoSco can be seen towards the top of the arena.

For functional performance testing we obtained *in vivo* results from a rat implanted as discussed in the following section, recording using both the RoSco system and a commercially available wired system; the Axona dacqUSB Recording System. Ideally these two recordings would be performed simultaneously, however recording in this manner introduced interference in both systems. Data for these comparisons were therefore recorded consecutively, Axona immediately followed by RoSco. Finally, as an example of real-world use of the wireless system, we recorded from a freely behaving rodent in a large, roofed outdoor enclosure located in Brisbane, Australia (Figure 4).

The Axona system was configured with 8000 gain, 16 bit at 48 kHz recording with the bandpass filter at 300–7000 Hz enabled. The RoSco system was configured for APs with 8000 gain, 8 bit at 20 kHz recording with a bandpass filter of 300–3000 Hz and LFPs with 1000 gain at 20 kHz recording with a bandpass filter of 3–3000 Hz.

### Electrophysiology procedure

Two adult male Long-Evans rats (∼370 g at surgery) were implanted with four tetrode Versadrives (Neuralynx) using standard surgical procedures as described in [17]. The electrodes were made of Nichrome (diameter 13 µm metal core; California Fine Wire) insulated with formvar. Electrode tips were electroplated with gold to achieve an impedance of 400–600 kΩ. The electrodes were implanted above the CA1 field of the left hippocampus. After a week of recovery each electrode was individually advanced along the dorso-ventral axis (60–80 µm) on a daily basis until neuronal activity was detected with a signal to noise ratio sufficient to allow for spike sorting. All indoor recordings were obtained while one animal was freely exploring a circular open-field (diameter: 0.8 m). The animals were then transferred to the outdoor facility and handled daily for 3 days. Following this period, recordings were obtained while the animals were foraging for one hour every day in a 3.5 m×2.5 m subsection of the outdoor recording enclosure and an elevated 0.8 m circular arena similar to that used indoors.

### Ethics statement

This study was performed in strict accordance with the recommendations in the Australian National Health & Medical Research Council Guidelines to promote the wellbeing of animals used for scientific purposes. The protocols were approved by the animals ethic Committee of The University of Queensland (Permit Number: QBI 049 11 NHMRC ARC).

## Results

This section describes comprehensive results for the RoSco. Sections V.A to V.E provide electrical characteristics: amplifier transfer function, CMMR, noise, and wireless performance. Section V.F provides sample neural recordings and comparisons to the Axona wired system.

### Filter and Gain Response

**Figure 5:**
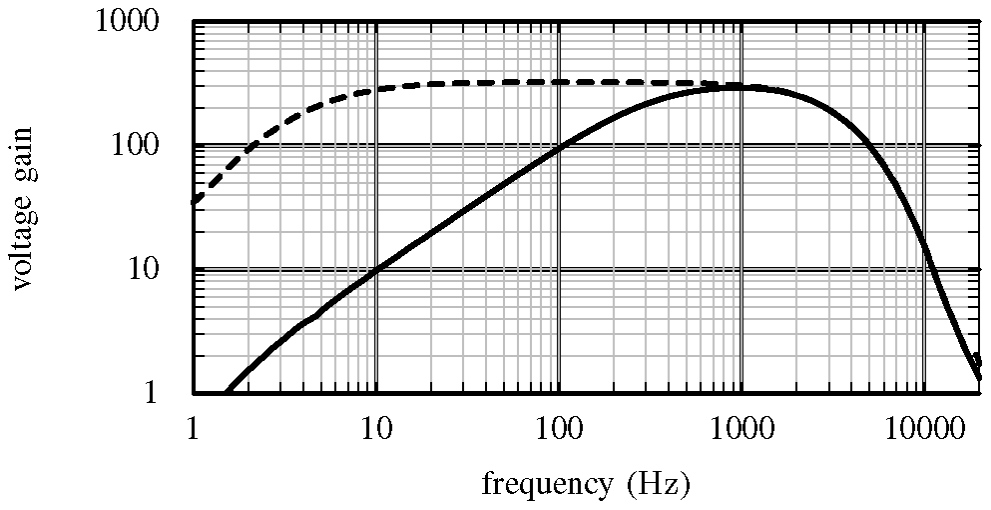
Measured transfer functions of the programmable bandpass filter. The filter can be configured for both a narrow-band mode for AP recording (solid line) and a wide-band mode for LFP recording (broken line). The output of this filter stage is further amplified by a programmable gain amplifier.

The signal conditioning stage described in III.A has a user-selectable filter and gain for LFP and AP recording. Figure 5 shows a measured transfer function for a representative electrode channel. The transfer function was measured using a frequency sweep technique [20]. The LFP and AP mode bandwidths are approximately 4–3000 Hz and 300–3000 Hz respectively. The output of this filter stage is further amplified in the microcontroller by a programmable gain amplifier.

### Common-mode rejection ratio

**Figure 6:**
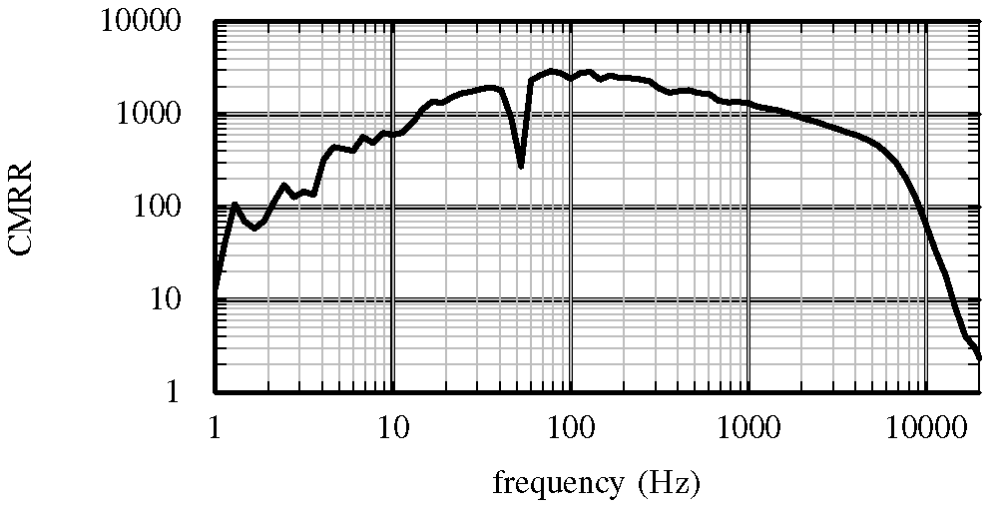
Measured common-mode rejection ratio. The CMRR measured for a representative channel and the selected reference. The rejection ratio is high over most of the band of interest.

The CMRR characterises the rejection of signals common to the channel of interest and the reference channel. A CMRR plot for a representative channel is shown in Figure 6. The downward spike in the CMRR at 50 Hz is a measurement artefact caused by interference from the Australian 50 Hz mains power used in the measurement instrumentation. The CMRR is in excess of 1000× (60 dB) over most of the band of interest. The CMRR is primarily limited by resistor tolerances of 0.1%.

### Noise

**Figure 7:**
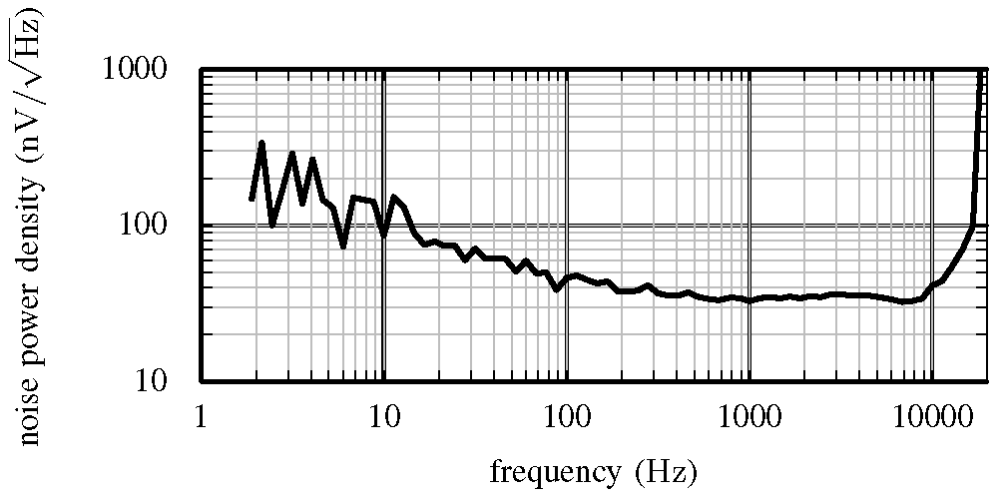
Measured noise power spectral density. This figure shows the measured noise power spectral density with the programmable band-pass filter programmed with the wide pass-band.

Figure 7 shows the noise power spectral density measured on one of the channels with the programmable band-pass filter span set to the 4–3000 Hz range. The noise power is approximately 40 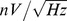 over the 300–3000 Hz pass-band used to record APs. This corresponds to an input referred noise of approximately 2.2 µV RMS. This noise level is insignificant compared to noise levels typically observed in neural signals acquired using tetrodes *in vivo*.

### Wireless Performance

#### Antenna radiation profile

**Figure 8:**
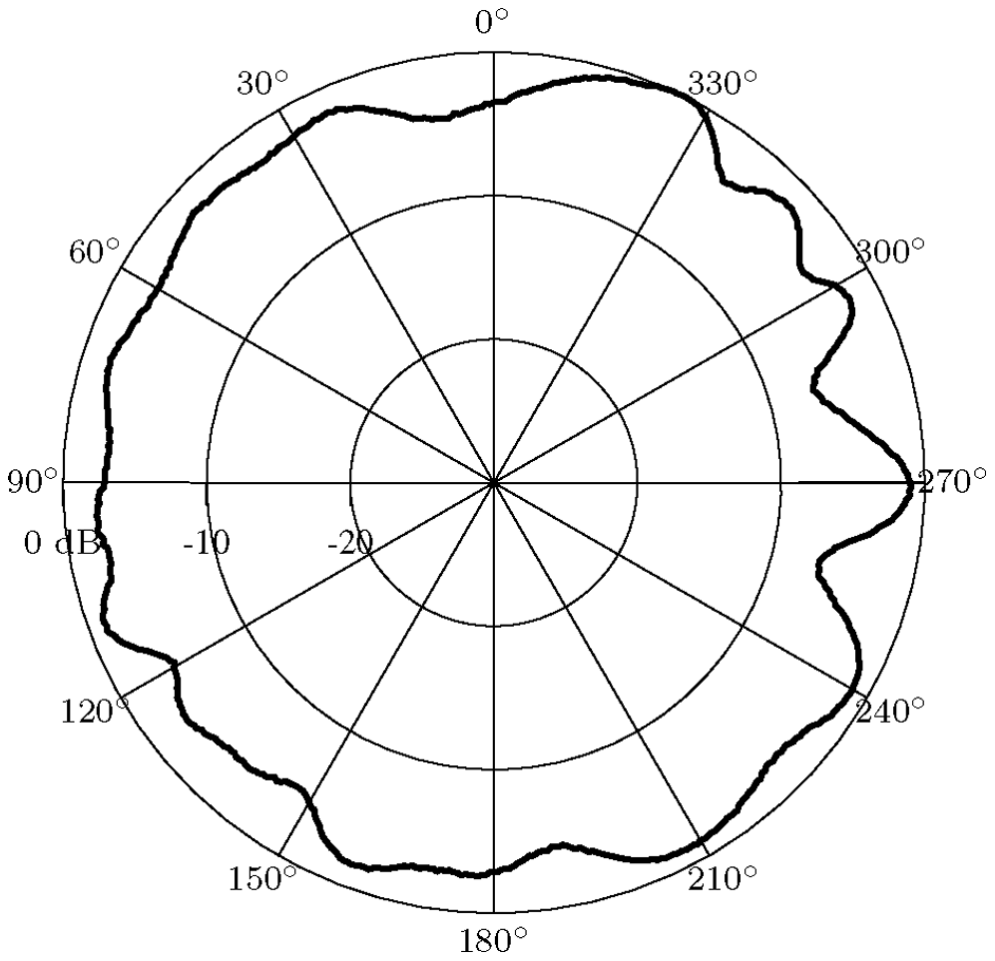
Antenna radiation profile. Measured horizontal plane radiation profile of the rat head-mounted module (vertical polarization).

As shown in Figure 8, the radiation from the rat head mounted module is excellent in all directions. The measured profile shows that an omnidirectional radiation pattern has been achieved with some ripple caused by the off-center antenna positioning and the square ground-plane geometry formed by the upper PCB. This omni-directional radiation pattern allows reliable wireless transmission regardless of the rodent's head direction relative to the base station.

The range of the system was evaluated with the RoSco placed at a number of distances, *d*, from the central point between two base station antennas placed 3 metres apart. This geometry is similar to the geometry that was used to record the neural signals for freely behaving rats in the outdoor arena. The omnidirectional base station antennas each had a gain of 5 dBi and were connected to the base station via 10 m cables which had a loss of 2.5 dB.

**Figure 9:**
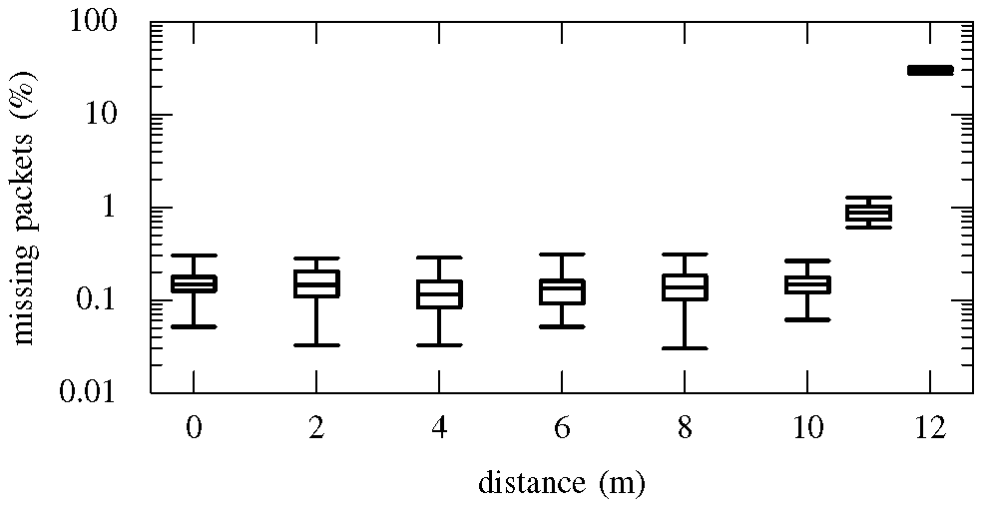
Wireless transmission error rate. Box plot of the error rate versus the distance between the rat module and the midpoint between the antennas. The figure shows that the practical limit for this wireless system with the given antenna configuration is 10

At each distance considered, the rate of missing packets was evaluated. Packets are automatically dropped by the transceivers when errors are detected, or when the signal is too weak. Errors are detected by first checking that the packet has a valid header, then by a CRC. The base station is phase-locked to the rat module, so the absence of packets is reliably noted. The percentage of lost packets was calculated by recording one second of data and counting the number of missing packet values compared to the total length of the recording. Thirty recordings were made at each distance in order to estimate the typical statistical range of missing packet rates. Figure 9 is a box plot showing the missing packet rate over a range of distances. (The whiskers indicate the minimum and maximum rates, the extent of the box corresponds to the interquartile range, and the horizontal bar indicated the median value.) The missing packet rate remains of the order of 0.1% from the zero distance position up to 10 m after which there is a sharp increase in the missing packet rate. This makes 10 m the practical limit for the system in the configuration tested, but longer range could be achieved with higher gain antennas and lower loss antenna cables.

#### Antenna diversity

**Figure 10:**
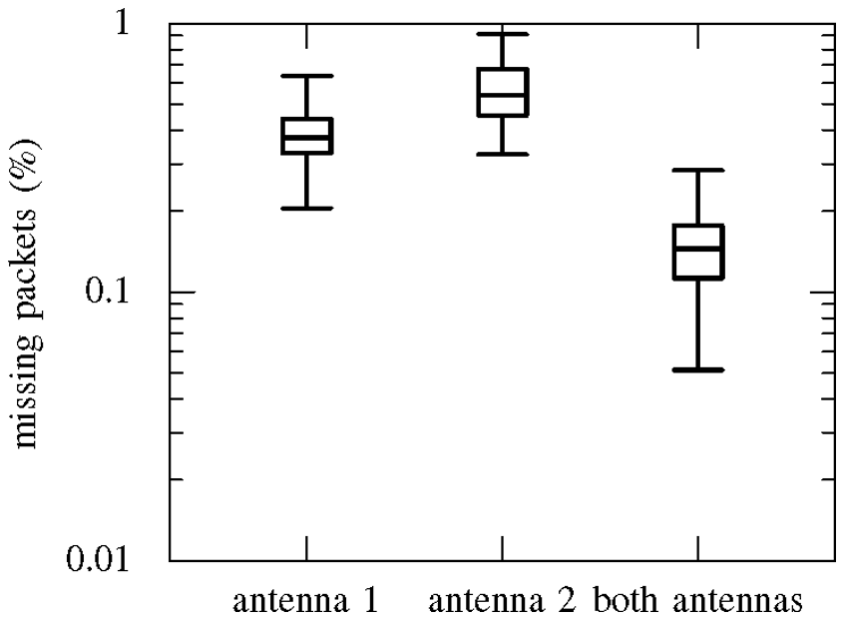
Wireless transmission error rate with diversity reception. Box plot of the error rate at 10

An example of the improvement in error rate that can be achieved using antenna diversity is given in Figure 10. The error rates were recorded under the same conditions as previously described at a fixed distance of 10 m, except that measurements were made with each of the antennas connected individually, then with both antennas connected. The median error rate drops from approximately 0.4–0.5% with one antenna connected (when diversity is effectively disabled) to less than 0.2% with both antennas connected.

### Battery Life

The battery life is approximately 1.5 hours which is sufficient for most experiments. It is important to note that battery life scales directly to the battery mass and size. For example, if the RoSco system needed to record for 3 hours then the mass would increase by 3 grams. Increasing the RoSco's mass by 3 grams would not significantly affect the rodent's mobility.

### Neural Recording

**Figure 11:**
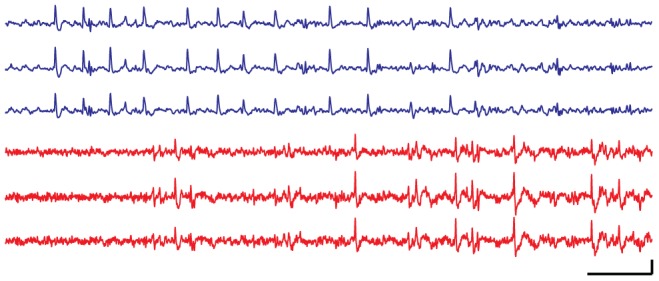
Sample neural waveforms from the RoSco and Axona system. 100(top three traces, blue) and Axona (bottom three traces, red). The RoSco signal has been digitally filtered with a high-order high-pass filter at 300 Hz. The increased detail of the Axona signal is due to the difference in sample rate: RoSco at 20 kHz and Axona at 48 kHz. Scale bars at bottom right are 10 ms and 50 µV for x-axis and y-axis respectively.

**Figure 12:**
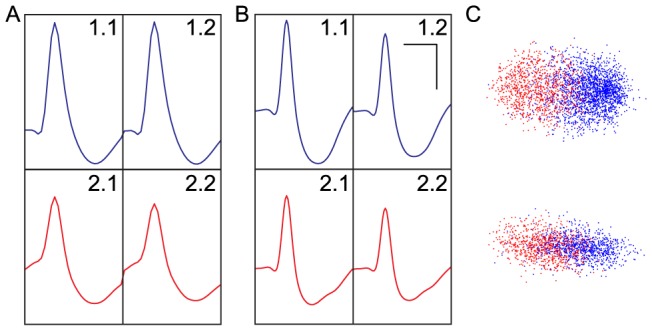
Comparison of characteristic unit spikes from the RoSco and Axona systems. (a, b) Waveforms were isolated from two units (blue – unit 1; red – unit 2) using (a) RoSco (RMS SNR range 2.0–3.1) and (b) Axona (RMS SNR range 1.8–2.8). Scale bars at top right are 500 µs and 50 µV for x-axis and y-axis respectively. (c) Example dimensions from the unit clustering, for the two units in a and b, obtained using the WaveClus package for the RoSco (top) and Axona (bottom) data. Both dimensions are unitless feature space.

The RMS SNR for detected spikes ranges from approximately 2.0 to 3.1 for RoSco, on average slightly greater than the RMS SNRs of 1.8–2.8 recorded from the Axona system. Examples of these raw signals are shown in Figure 11. After spike detection and clustering, similar units are visible on both the wired and wireless systems (Figure 12). These units are more similar across systems (Axona vs RoSco) than within systems (unit 1 vs unit 2), as measured by their correlations (Axona unit 1 to RoSco unit 1 = 0.851, Axona unit 2 to RoSco unit 2 = 0.855, Axona unit 1 to Axona unit 2 = 0.723, RoSco unit 1 to RoSco unit 2 = 0.722). The difference in the same units across systems (correlations of 0.85 instead of 1) is due to the lower filter cut-off in the Rosco system causing the spikes to be somewhat broader. The small differences in correlations between the same units across systems (0.855–0.851 = 0.004) and between different units in the same system (0.723–0.722 = 0.001) indicates that these differences are systematic and that the spike detection and sorting has not been affected by RoSco's lower bit depth and sampling rate; a result which is supported by theory (i.e. the Nyquist theorem and the required bit depth given the expected SNR).

**Figure 13:**
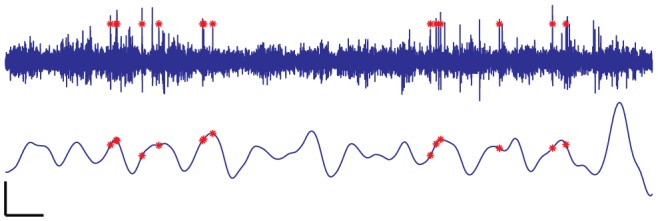
Electrical recording and identified spike times from two units. One unit from the dentate gyrus is shown with red stars in: (a) the raw trace and (b) a theta-filtered 4-12 Hz LFP. Unit activity appears more often on particular phases of the theta cycle, as expected from many earlier hippocampal studies [19], [20]. Scale bars at bottom left are 100 ms for x-axis and 50 µV/250 µV for top/bottom y-axes.

Neural implants were in the dentate gyrus and the CA1 hippocampal subregion (see Section 4), where unit activity is often correlated with a 6–10 Hz oscillation in the LFP [21], [22]. Low frequencies are attenuated by the band-pass filter to only a fraction of one percent of the total signal power in the spike frequency range. The remaining low frequency power is sufficient to be detected and isolated with a low-pass filter, but is too small to have a material effect on spike detection. Analysis of the theta-band LFP shows that spikes are correlated with certain phases of theta (Figure 13). Note that all filters are applied bi-directionally so there is no net phase distortion.

**Figure 14:**
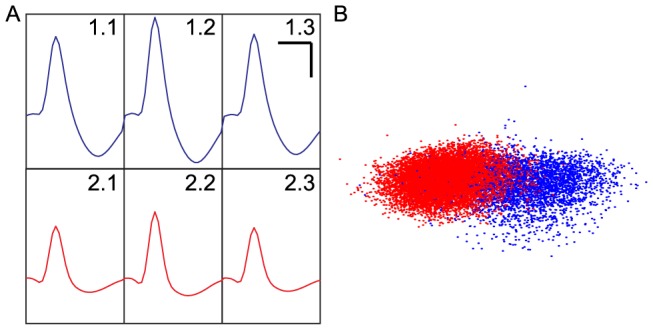
Characteristic unit spikes captured by RoSco in the outdoor enclosure. The unit spikes were taken from three wires on one tetrode. (a) The characteristic spike waveforms of two units. SNRs (RMS) are between 1.3 and 2.8 depending on the unit and the wire. Scale bars in top right are 500 µs and 50 µV for x-axis and y-axis respectively. (b) Example dimensions from the unit clustering obtained using the WaveClus package. Both dimensions are unitless feature space.

One of the primary purposes of the wireless system is to allow animals to explore larger, more natural environments. Wireless samples were collected while a rat foraged in a subsection of a large 5 m×7 m roofed outdoor cage (shown in Figure 4), for approximately 45 minutes. Unit activity was similar to that collected indoors (Figure 14).

## Discussion

We have described the design and operation of RoSco, a wireless telemetry recording system designed for single unit and field potential neural recordings from freely moving animals. This telemetry system has quantifiable fidelity through the use of digital transmission, and has the minimal set of expected user-configurable options. We demonstrate that the recording, and signal-to-noise ratios and action potential traces are comparable to an existing commercial wired system. This telemetry system extends current available tools available for experimental neuroscience into new areas, allowing recording and tracking of rodent navigation in natural outdoor environments.

The design of small lightweight recording systems for rodent studies required several tradeoffs. See Table 1 for a comparison between RoSco and state-of-the-art existing wireless solutions. There are many other analog wireless systems, including several commercial solutions, however they have similar performance to the analog wireless modules shown in the table. The choice between analog or digital design is one of the most fundamental decisions for a wireless system. It affects all other aspects of the design, including the size, weight, and power requirements of the device, the ability to control the device remotely, which affects user configuration, and the ability to quantify the quality of the transmitted signal.

**Table 1:** Comparison between RoSco and existing systems.

	RoSco	Fan et al.	Szuts et al.	HermesD*
Transmitter type	Digital	Analogue	Analogue	Digital
Battery life	1.5 hrs	6 hrs	6 hrs	Not reported
Bandwidth	4–3000 Hz or 300–3000 Hz	0.8–7000 Hz	10–4000 Hz	Not reported
Size	35×35×35 mm	2.2 cm^3^	100 cm^3^	38×38×51 mm
Mass	22 g	4.5 g	52 g	Not reported
Gain	500–32,000×	800×	1800×	Not reported
Configurable gain	7 options	No	No	No
Input referred noise	2.2 µV RMS	10 µV RMS	Not reported	Not reported
Range	10 m	4 m	60 m	>20 m
Channels	14/16 channels @ 20 kHz @ 8 bit OR 7/8 channels @ 40 kHz @ 8 bit	Variable	64 channels @ 20 kHz	32 channels @ 30 kHz @ 12 bit

*Note that the HermesD system is designed for primates.

Analog recordings are familiar to all electrophysiologists, since most commercial neural recording systems are wired and transmit the neural signals over the tethered link in analog form (e.g. Axona, Plexon, Neuralynx). Similar to wired systems, the majority of wireless systems use analog transmission. Such systems are smaller and lighter than comparable digital systems and power consumption is significantly lower for equivalent data transmission, as demonstrated in existing commercial analog wireless system solutions [15], [23]. Transmission fidelity in such analog systems is typically taken for granted and not quantified, since recordings are made in shielded rooms with careful control of all sources of RF noise, enabling high SNR. However, even in wired systems in such environments, noise can be introduced through the tether itself, which can act as an antenna, or the commutator that enables the animal to move without tangling the cable.

Outside shielded environments, RF noise cannot be controlled and for signals to be trusted, signal quality needs to be monitored as a matter of routine. None of the analog systems published to date (wired or wireless) have methods for quantifying the fidelity of the signals as they are transmitted.

Our decision to digitize before transmission followed directly from the requirement that RoSco be functional in an uncontrolled outdoor-like environment, where interference is prevalent, and needs to be routinely identified and managed. RoSco's digital system enables transmission without error under ideal conditions, and with routine reporting of dropped packets when noise interferes with signal transmission.

Another design issue that impacts on confidence in signal quality is whether spikes are processed on the headstage prior to transmission or whether full waveforms are transmitted allowing offline analysis. While primitive spike detection can be achieved using a manual thresholding technique, current leading automatic detection algorithms rely on long-term signal characteristics, requiring the full waveforms in post-processing [24]. Full waveforms require more bandwidth, however they are essential for the confidence that offline computation provides in particular while recording in new experimental paradigms.

Per-channel bandwidth is determined by the sampling rate and the bit-depth. Theoretically, the required sampling rate is determined by the high frequency cut-off of the signal. The Nyquist theorem defines that the sampling rate with perfect filters and transformations needs only be twice the maximum frequency [25]. In practice filters are not perfect and higher sampling rates are required to ensure aliased signals are acceptably small. The required bit-depth is dependent on the signal-to-noise ratio of the signal. In the case of action potentials, the signal is the action potential waveform, and the noise is all other electrical activity.

Theoretical data transmission rates of existing systems range from a few hundred Kbps [9] to 90 Mbps [11], though experimentally-verified implementations to date have been limited to 24 Mbps [8]. Designs such as [4] were intended to enable full configurability of these factors, albeit with increased hardware complexity. RoSco's setting of 20 kHz at 8 bits per sample is sufficient for its filter cut-offs and the expected signal to noise ratio of APs. Beyond this theoretical argument, 48 kHz is a typically sampling frequency of wired systems. For comparison studies, RoSco provides a setting to double the sampling rate to 40 kHz at the cost of halving the number of recorded channels. However in preliminary studies, spike sorting with the 48 kHz signal did not produce any noticeable benefit to the 20 kHz recordings. Recently, Multichannel Systems have released the W-System range of digital wireless neural recording systems, although without the range of configurable settings available in our system.

The useability set of design decisions for RoSco impacts on practical experiments, in particular, the configurability of the system. Commercial wired systems support a high level of reconfiguration needed for extended studies using animals with chronically implanted electrodes. A wire in a region with little activity is typically selected as a reference for the other wires, but the reference can change over time, as electrodes can move with respect to the cells they record, requiring higher or lower levels of gain, with new cells appearing and previously recorded ones disappearing and disabling needed for broken wires.

Online configuration is supported in RoSco. Reconfiguration of parameter settings after the head stage has been attached to the animal enables online control of settings for gain, bandwidth, grounding, and reference. In addition four LEDs on the headstage can be individually configured, enabling tracking and also wireless control of synchronization signals during an experiment. These features are particularly useful for practical experiments, making configuration of the module efficient and enabling adjustment to the neural conditions in the moment. No other system (digital or analog) matches the breadth of online configuration supported by RoSco. In fact, most analog systems are limited to simplex communication that cannot support online configuration at all.

In summary, criteria for wireless telemetry systems result in a spectrum of designs: RoSco is a digital system enabling quantifiable high fidelity, and user configuration. RoSco's module is heavier than analog systems but still of suitable size and mass for mounting on the head of rat. A single battery provides 1.5 hours of operation, sufficient for outdoor foraging experiments. Additional batteries can be added with linear increase in performance. For example, recording time can be increased to three hours by using two batteries, and would increase the mass to 25 g and the height of the head mount by 6 mm. This would still be suitable for use on a rodent's head.

The reported 10 m range for 0.1% loss is sufficient for a wide range of experiments in large indoor and outdoor environments. The antenna was designed for general purpose use and hence has an omni-directional radiation pattern. It proved effective for the studies in this paper and will be used in future large arena foraging studies. However, it is important to note the radial range is dependent on the antenna design. A different antenna design, with higher gain or different radiation profiles, could extend the range further.

Natural social interactions require animals to have multiple degrees of freedom of movement which are impossible with tethered systems. For future studies involving social interactions, up to 31 RoSco systems can be used simultaneously, each controlled by an independent interface.

The RoSco is designed for use as part of a larger system that records from multiple rodents and tracks their motion. Motion tracking is via LEDs on the module and an overhead camera system. The base station transmits the neural waveform in the standard USB audio protocol that can use standard methods and containers for combining and playing multiple sources of audio and video, such as for movies. These methods include the ability to maintain synchronisation of the streams during data loss.

## Conclusion

Wireless telemetry systems are as varied as the empirical studies used to investigate neural signals. The RoSco telemetry system was specifically designed for outdoor rodent navigation and behavioural studies, with primary design criteria being quantifiable fidelity and usability. The system has been demonstrated to be empirically useful in animal studies, with 8 bit, 20 kHz signals successfully providing full waveform recordings amenable to spike sorting.

Digital transmission in large scale environments resulted in low noise over a 10 metre range. The small head mounted module was well-tolerated by the animals, enabling freedom of movement beyond anything possible with a tethered system, with battery life enabling experiments to extend over 1.5 hours. The user-configurable settings for remote gain control, LFP filtering, reference selection and channel muting enabled an experimental workflow similar to commercial wired systems.

Wireless systems will continue to expand the range of environments in which recordings can be made, and extend the possibilities for studies in natural and enriched environments. RoSco provides a useful addition to the current range of wireless systems, providing new capabilities compared to published and commercially available modules and enabling new science in the field of small animal behaviour.
